# Constructing the resting state structural connectome

**DOI:** 10.3389/fninf.2013.00030

**Published:** 2013-12-05

**Authors:** Olusola Ajilore, Liang Zhan, Johnson GadElkarim, Aifeng Zhang, Jamie D. Feusner, Shaolin Yang, Paul M. Thompson, Anand Kumar, Alex Leow

**Affiliations:** ^1^Department of Psychiatry, University of IllinoisChicago, IL, USA; ^2^Laboratory of Neuro Imaging, Department of Neurology, University of CaliforniaLos Angeles, CA, USA; ^3^Department of Electrical and Computer Engineering, University of IllinoisChicago, IL, USA; ^4^UCLA Semel Institute for Neuroscience and Human Behavior, University of CaliforniaLos Angeles, CA, USA; ^5^Department of Bioengineering, University of IllinoisChicago, IL, USA; ^6^Community Psychiatry AssociatesSacramento, CA, USA

**Keywords:** connectivity, fMRI, major depression, multimodal, neuroimaging

## Abstract

**Background:** Many recent studies have separately investigated functional and white matter (WM) based structural connectivity, yet their relationship remains less understood. In this paper, we proposed the functional-by-structural hierarchical (FSH) mapping to integrate multimodal connectome data from resting state fMRI (rsfMRI) and the whole brain tractography-derived connectome.

**Methods:** FSH first observes that the level of resting-state functional correlation between any two regions in general decreases as the graph distance of the corresponding structural connectivity matrix between them increases. As not all white matter tracts are actively in use (i.e., “utilized”) during resting state, FSH thus models the rsfMRI correlation as an exponential decay function of the graph distance of the rsfMRI-informed structural connectivity or rsSC. rsSC is mathematically computed by multiplying entry-by-entry the tractography-derived structural connectivity matrix with a binary white matter “utilization matrix” U. U thus encodes whether any specific WM tract is being utilized during rsFMRI, and is estimated using simulated annealing. We applied this technique and investigated the hierarchical modular structure of rsSC from 7 depressed subjects and 7 age/gender matched controls.

**Results:** No significant group differences were detected in the modular structures of either the resting state functional connectome or the whole brain tractography-derived connectome. By contrast, FSH results revealed significantly different patterns of association in the bilateral posterior cingulate cortex and right precuneus, with the depressed group exhibiting stronger associations among regions instrumental in self-referential operations.

**Discussion:** The results of this study support that enhanced sensitivity can be obtained by integrating multimodal imaging data using FSH, a novel computational technique that may increase power to detect group differences in brain connectomes.

## Introduction

Modern imaging techniques have allowed us to study the human brain as a complex system by modeling it as a network. A brain connectivity network, also called a connectome (Sporns et al., 2005), consists of nodes (gray matter regions) and edges. Edges can represent white matter tracts in structural networks or correlations between two BOLD time series in functional networks.

In recent years, substantial research efforts have been directed toward understanding the brain at rest using resting state functional MRI (rs-fMRI). Several studies have utilized sophisticated mathematical and statistical tools to investigate the functional connectome from rs-fMRI data (Biswal et al., 1997). The “default mode network” (DMN) is a resting-state network theorized to reflect an individual's focus on internal tasks such as daydreaming, envisioning the future, retrieving memories, and gauging others' perspectives. The DMN tends to negatively correlate with brain systems responsive to external signals. Anatomical regions involved include the medial temporal lobe, the medial prefrontal cortex, and the posterior cingulate cortex (Buckner et al., 2008), along with the adjacent precuneus (Zhang and Li, 2012) and the parietal cortex.

The DMN is an example of one relatively well-characterized network, among many overlapping networks that subserve different functions. Delineating these functional connections may therefore be challenging, based on the complexity of the brain. Structural white matter connectivity patterns, however, may provide a framework for understanding relevant functional relationships between regions in a network, based on direct and indirect anatomical connections. This may aid in determining information available as outputs from certain regions and its inputs and potential influence on other regions (Saygin et al., 2011). An approach using structural to functional mapping could utilize a combination of DTI-tractography to estimate brain white matter connectivity and fMRI to estimate the neuronal activity coupled to blood flow changes in anatomical regions that comprise nodes of the network.

There have been several structural to functional mapping approaches described in the literature. While some have focused on specific but limited regional activation patterns (Johansen-Berg et al., 2004; Saygin et al., 2011), other models describe functional connections within regions comprising larger networks or systems (Passingham et al., 2002; Honey et al., 2009; Deligianni et al., 2010; Skudlarski et al., 2010; Chulwoo et al., 2011; Varkuti et al., 2011; Ng et al., 2012). Of note, these studies reviewed here all considered structural connectivity to be static, unlike their functional counterparts. However, it is highly unlikely that white matter tracts are static in relation to the brain's different functional states. Indeed, white matter tracts can be in use or engaged when the brain is performing certain tasks but disengaged during other tasks (e.g., the white matter structure subserving the DMN will be relatively disengaged when the brain is responding to external signals). In addition, some of these previously published techniques rely on statistical methods based on linear modeling, however the relationship between structural and functional connectivity may be non-linear (Deligianni et al., 2010). In other studies, sparse Gaussian graphical modeling (SGGM) is used for multimodal integration (Ng et al., 2012). There, the authors proposed to merge functional and tractography-derived structural data by casting functional connectivity estimation as a sparse inverse covariance learning problem. As functional connections with less anatomical support (i.e., fewer streamlines or fiber tracts) were more penalized via an L1 type penalty term, the resulting functional connection patterns could thus be considered structural connectivity-informed.

Here, in contrast to such SGGM models, we reversely consider functional connectivity-informed structural connectivity, thus arguing that information from fMRI can be used to infer the underlying pattern of white matter engagement specific to the brain's state at the moment of the fMRI. To address that not all white matter tracts are in use or engaged during fMRI, we will extend and adapt the functional by structural hierarchical mapping (FSH) technique, a novel framework recently proposed by our group (Leow et al., 2012) in order to estimate white matter engagement or utilization patterns that generate the functional connectome from rs-fMRI data, using structural networks derived from DTI-tractography. The resulting connectome, which we term the resting-state informed structural connectome (rsSC), encodes the structural network that underlies and facilitates the observed rs-fMRI correlation connectome. Moreover, we may detect group differences in rsSC by investigating and comparing their community or modular structures. To this end, we utilized PLACE (path length associated community estimation) (GadElkarim et al., 2013) and detected altered rsSC community structure in depressed subjects relative to controls.

## Materials and methods

### Subject selection

7 healthy comparison (HC, age: 65.6 ± 8.12, 4 males) and 7 late-life depressed (LLD, age: 60.7 ± 2.92, 4 males) subjects, were recruited via community outreach (e.g., newspaper, radio, and television advertisements) and relevant outpatient clinics. The inclusion criteria for all subjects were 55 years of age and older, medication-naive or anti-depressant free for at least 2 weeks (in the case of our depressed subjects) and no history of unstable cardiac or neurological diseases. The exclusion criteria included: schizophrenia, bipolar or any psychotic disorders; history of anxiety disorder outside of major depressive episodes; history of head trauma; history of substance abuse; contraindications to MRI such as metal implants. This study was approved by the University of Illinois-Chicago Institutional Review Board, and written informed consent was obtained from each participant. There were no significant differences in age (*t* = 1.49, *p* = 0.18) and gender distribution (χ^2^ = 0, *p* = 1) between subject groups. LLD subjects had a mean HAM-D score of 20 ± 3.7.

All eligible subjects were assessed by a trained research assistant with the Structured Clinical Interview for Diagnostic and Statistical Manual of Mental Disorders, Fourth Edition (DSM-IV). The severity of depression was quantified by a board-certified/board-eligible psychiatrist (AK or OA) using the 17-item Hamilton Depression Rating Scale (Hamilton, 1960). At the time of enrollment, depressed subjects met DSM-IV criteria for MDD and required a score of 15 or greater on the HAM-D.

### MRI acquisition

Brain MRI were acquired on a Philips 3.0T Achieva scanner (Philips Medical Systems, Best, The Netherlands) using an 8-channel SENSE (Sensitivity Encoding) head coil. Participants were positioned comfortably on the scanner bed and fitted with soft ear plugs; foam pads were used to minimize head movement. Participants were instructed to remain still throughout the scan. High resolution three-dimensional T1-weighted images were acquired with a MPRAGE (Magnetization Prepared Rapid Acquisition Gradient Echo) sequence (field of view: FOV = 240 mm; 134 contiguous axial slices; TR/TE = 8.4/3.9 ms; flip angle = 8°; voxel size = 1.1 × 1.1 × 1.1 mm). Resting-state data were acquired with the following parameters: Single-shot gradient-echo EPI sequence, TR/TE = 2000/30 ms, Flip angle = 80°C, EPI factor = 47, FOV = 23 × 23 × 15 cm^3^, in-plane resolution = 3×3 mm^2^, slice thickness/gap = 5/0 mm, slice number = 30, SENSE reduction factor = 1.8, NEX = 200, total scan time = 6:52. Subjects were instructed to keep their eyes close and “not think of anything in particular”. DTI images were acquired using single-shot spin-echo echo-planar imaging (EPI) sequence (FOV = 240 mm; voxel size= 0.83 × 0.83 × 2.2 mm; TR/TE = 6,994/71ms; Flip angle = 90°C). Sixty seven contiguous axial slices aligned to the anterior commissure–posterior commissure (AC-PC) line were collected in 32 gradient directions with b = 700 s/mm^2^ and one acquisition without diffusion sensitization (B0 image). Parallel imaging technique was utilized with factor at 2.5 to reduce scanning time to approximately 4 min.

### Data preprocessing

Structural connectomes were generated using a pipeline which integrates multiple image analysis techniques and has been reported elsewhere (GadElkarim et al., 2012; Leow et al., 2012). In brief, DW images were eddy current corrected using the automatic image registration (AIR) tool embedded in DTIStudio software (http://www.mristudio.org) by registering all DW images to their corresponding b0 images with 12-parameter affine transformations. This was followed by the computation of diffusion tensors and deterministic tractography using the DTIStudio program. T1-weighted images were used to generate label maps using the Freesurfer software (http://surfer.nmr.mgh.harvard.edu). Brain networks formed by the 82 cortical/subcortical gray matter regions were generated using an in-house program in Matlab by counting the number of fibers connecting each pair of nodes.

Functional connectomes were generated using the resting-state fMRI toolbox, CONN (http://www.nitrc.org/projects/conn;Whitfield-Gabrieli and Nieto-Castanon, 2012). In brief, raw EPI images were realigned, co-registered, normalized, and smoothed before analyses. Confound effects from motion artifact, white matter, and CSF were regressed out of the signal. Using the same 82 labels as the structural brain networks, functional brain networks were derived using pairwise BOLD signal correlations, which were then converted to *z* scores using Fisher's r-to-z transformation.

### Functional by structural hierarchical (FSH) mapping for constructing rsSC

Several assumptions and simplifications are needed in order to perform FSH mapping (Leow et al., 2013). However, in order to generalize FSH to construct rsSC, several modifications are necessary, which we outlined step-by-step as follows:

1. Higher level of rs-fMRI correlations will be considered evidence of strong structural interactions between two regions (either through direct or indirect structural connections in the corresponding DTI-derived structural network)
2. We observe that in general the level of rs-fMRI correlation between two regions decreases as the graph distance of the DTI-derived structural connectivity matrix increases between them. FSH further assumes that such a relationship is mathematically an exponential decay:
  (1) $$level\;of\;rsfMRI\;correlation\;between\;i\;and\;j\approx e^{-kf_{i,j}\left(D\right)}$$
  Here, k a rate constant to be estimated, D the DTI-derived structural connectivity matrix for the same subject, and functional *f* denotes the mapping of a brain connectivity matrix to its graph distance matrix (i.e., each entry denotes the shortest graph distance between node pairs). Here, *f* is numerically obtained by applying the Dijkstra algorithm to the entry-wise inverse of D (since stronger structural connectivity translates to shorter distance, edge lengths are then usually assumed to be the inverse of connectivity strengths).
3. As in the original formulation of FSH, the presence of an edge connecting any node pair in the structural connectivity matrix predicts the existence of neuroanatomical white matter connections between regions, which may or may not be actively utilized when the brain is in the resting state. In order to reduce the mathematical complexity in modeling and parameter fitting, FSH assumes an all-or-nothing edge utilization (i.e., an edge is either utilized or not at all). A connection between node m and n is considered “utilized” if including the anatomical connection between them better predicts the overall resting state fMRI correlation. This is thus mathematically represented by a binary utilization matrix U (i.e., if *U*_*(i, j)*_ = 1, then the WM structural connection between nodes i and j are utilized in the resting state; zero otherwise)
4. FSH now hypothesizes that a direct mathematical relationship can be established, for each node pair, between the level of rs-fMRI correlation and the modulated graph distance between the two nodes for the DTI-derived structural network according to the utilization matrix, via the following modified exponential decay equation:
  (2) $$level\;of\;rsfMRI\;correlation\;between\;i\;and\;j=e^{-kf_{i,\;j}\left(U^{{}^{\circ}}D\right)}+\varepsilon$$
  Here U is the utilization matrix (same dimension is D), ° the Hardamard entry-by-entry multiplication operator between two matrices of the same dimensions, ε the fitting error (assumed to be normally distributed).
  Note the above exponential functional dictates that rsfMRI correlations exponentially decay with increasing modulated graph distance, and that when the modulated graph distance between two nodes approaches infinity (i.e., the nodes are far away from each other), the corresponding rsfMRI correlation as expected approaches zero (by contrast, if two nodes are infinitesimally close, the rsfMRI correlation is 1).
5. For subjects in the same diagnostic group, we fit on the group level, by minimizing the sum of squared differences between the observed and the predicted rsfMRI correlations for all node pairs (both are z-transformed), such that the group utilization matrix U is assumed to capture certain characteristics unique to this group. To this end, the resting state informed structural connectome is mathematically*U*° *D*. Mathematically, the minimization problem for solving group-wise U is as follows (the superscript n denotes subjects in the same diagnostic group; in this study n ranges from 1–7).
  (3) $$U=argmin\sum_{n}\sum_{i,\;j}{\left(\left|rsfMRI\;correlation_{i,\;j}^{n}\right|-e^{-k^{n}f_{i,\;j}\left(U^{{}^{\circ}}D^{n}\right)}\right)}^{2}$$
6. To solve k (unique to each subject) and the utilization U (shared for each diagnostic group), we closely follow the original FSH formulation by alternating between the estimation of k and U. When fitting U, we used simulated annealing by randomly picking one element in U and changing its value (between 0 and 1; the initial value of U was set to one for all its entries, thus indicating all edges were utilized and U°D simply returned the original structural connectivity matrix D). The acceptance criterion determined whether the new state was accepted from the current state by applying the following decision rule with respect to an artificial cooling temperature (c).

(4) $$\begin{array}{l} probability\;of\;accepting\;a\;proposed\;new\;state=\\ \{\begin{array}{ll} 1 & \;if\;the\;new\;state\;yields\;a\;lower\;fitting\;residual\\ \exp\left(-\frac{fitting\;residual\;increase}{c}\right) & \;if\;the\;new\;state\;yields\;a\;higher\;fitting\;residual \end{array} \end{array}$$

This perturbation was repeated and the temperature gradually decreased until the solution space was adequately sampled and the global minimum reached.

To assess the goodness of fit of FSH mapping, we calculated the correlation between the observed rs-fMRI z-scores and the predicted rs-fMRI z-scores according to the exponential decay function, both without (**Equation 1**) and with the utilization matrix (**Equation 2**). The effect of fitting utilization matrix was then tested by comparing the two groups of correlation coefficients using the Fisher's r-to-z transform.

### Modular structure using place (path length associated community estimation)

After FSH mapping, we constructed the rsSC separately for the depressed and the control group, by forming the product *U*° *D* using group-specific utilization matrix and group-average structural connectivity matrix. We then used the PLACE (path length associated community estimation) framework presented in (GadElkarim et al., 2012, 2013) to assess potential group differences for structural connectome (DTI-derived) alone, the functional connectome (rs-fMRI-derived) alone, and the resting state informed structural connectome. PLACE is a novel technique designed to detect and compare hierarchical modular or community structure alterations between two groups of brain networks based on shortest path lengths, and has been shown to be advantageous when compared to the modularity metric Q (Newman and Girvan, 2004; Blondel et al., 2008).

To summarize, in PLACE community structures are first extracted in the form of top-down hierarchical binary trees via the maximization of a path-length dependent metric Ψ^*PL*^, defined as the difference between the average inter-community path-length (inter_*PL*_) and the average intra-community path-length (intra_*PL*_), for two communities *C*_1_ and *C*_2_, Ψ^*PL*^ is mathematically defined as:

(5) $$\Psi^{\text{PL}}={\text{inter}}_{\text{PL}}^{C_{1},C_{2}}-\frac{1}{2}\left({\text{intra}}_{\text{PL}}^{{\text{C}}_{1}}+{\text{intra}}_{\text{PL}}^{{\text{C}}_{2}}\right)$$

Where

(6) $${\text{inter}}_{\text{PL}}^{C_{i},C_{j}}=\frac{\sum_{n,\in C_{i};m\in C_{j}}d_{nm}}{N_{i}N_{j}}{\text{intra}}_{\text{PL}}^{{\text{C}}_{\text{i}}}=\frac{\sum_{n,m\in C_{i};n>m}d_{nm}}{\left(N_{i}^{2}-N_{i}\right)/2}$$

where *N_i_* is the number of nodes in community *C_i_*, *d_nm_* is the shortest path length (i.e., graph distance) connecting nodes n and m.

To quantify node-level community differences, PLACE uses the *scaled inclusivity* metric *V* (Steen et al., 2011) in which a nodal consistency vector of length equal to the number of nodes in the network (82 in our case) is generated to compare nodes in a *test* tree (i.e., an individual subject's tree) to nodes in a *reference* tree. Mathematically, for each node *k* belonging to communities *C_p_* and *C_q_* in the *test* and *reference* trees respectively, *V* is defined as; *V(k)* = (*N_c_*)^2^/*N_p_N_q_*, where *N_c_* is the number of common nodes between *C_p_* and *C_q_*.

In order to examine group differences in community structures at the nodal level, one group of networks is chosen as the reference and PLACE generates the *reference* tree by extracting the community structure corresponding to the reference group's mean connectivity matrices (using node-wise averaging). Next, all individual subjects' trees are compared to the *reference* tree, yielding the node-level scaled inclusivity metric, *V*. For each node, 2-sample *t*-tests for *V* are then used to detect differences in the community structure on the nodal level (relative to the reference group), followed by multiple comparisons correction conducted using the false discovery rate (FDR) (Benjamini and Hochberg, 1995).

## Results

**Figure 1:**
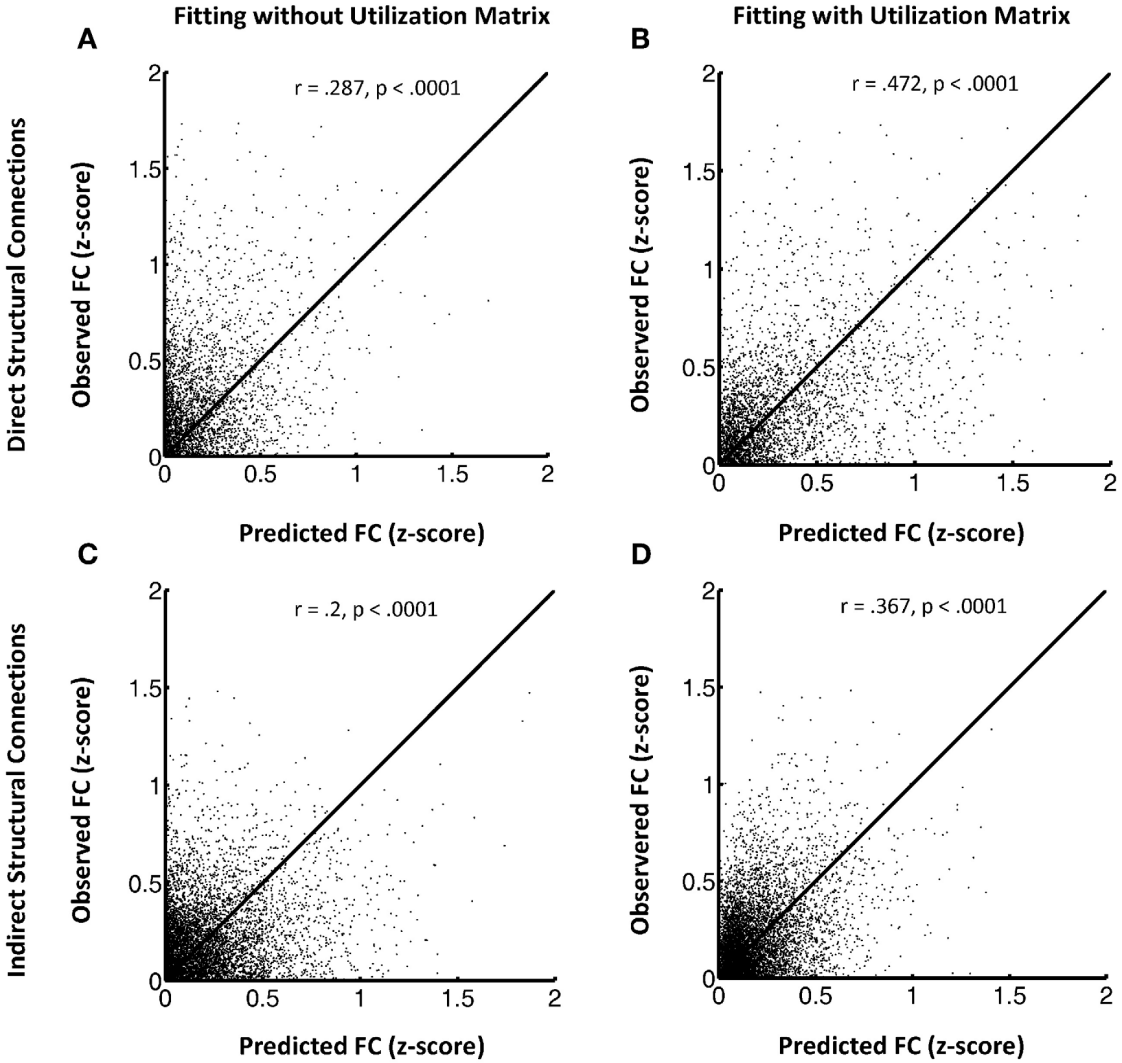
**(A–D)** This shows the FSH mapping results for all node pairs, collected from all subjects in the HC group for region pairs with direct structural connections **(A and B)** versus those without direct structural connections **(C and D)**. Left panels display the model fitting without the utilization matrix U and the right panels show fitting with the utilization matrix in the proposed exponential decay model. The y axis indicates observed resting state fMRI correlation values and the x axis the predicted resting state fMRI correlation values.

Figure 1 shows the FSH mapping results, which confirmed improved fitting with the additional inclusion of the utilization matrix U in the exponential decay function, in the HC group (Figures 1A–D) by plotting the observed rsfMRI correlations values against the predicted values. Here, we break down the results for HC subjects into two groups: region pairs with direct structural connections between them (**1A and 1B**) versus pairs without direct structural connections (i.e., only indirect structural connections; **1C and 1D**). Overall, the proposed FSH-exponential decay model significantly improved the correlation between rsfMRI and structural connectivity as all data points moved toward the line *x* = *y* after fitting (direct: *z* = −8.7, *p* < 0.0001; indirect *z* = −10.3, *p* < 0.0001). The pattern also occurred for LLD subjects (direct: *r* = 0.246 (without fitting *U*), *r* = 0.509 (with *U*), *z* = −12.3, *p* < 0.0001;indirect: *r* = 0.188 (without fitting *U*), *r* = 0.267 (with *U*), *z* = −4.8, *p* < 0.0001). Unsurprisingly, node pairs with direct structural connections exhibited stronger associations with rsfMRI correlations compared to those with only indirect connections.

**Figure 2:**
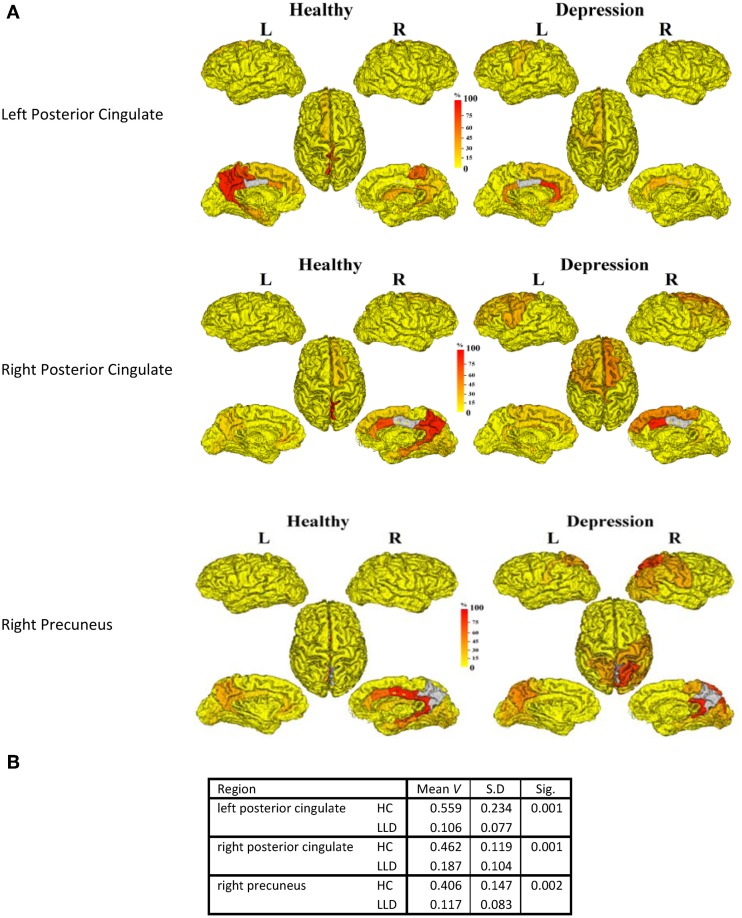
**(A)** The following three regions (in gray) exhibit significant group differences after FDR correction:. 1. Left posterior cingulate. 2. Right posterior cingulate. 3. Right precuneus. The frequency of shared community membership for these regionsin HC and LLD. 100% indicates all seven subjects from the same diagnostic group have this region assigned to the same community as the gray region. **2B:**. The mean consistency values (V) for each region.

**Figure 3:**
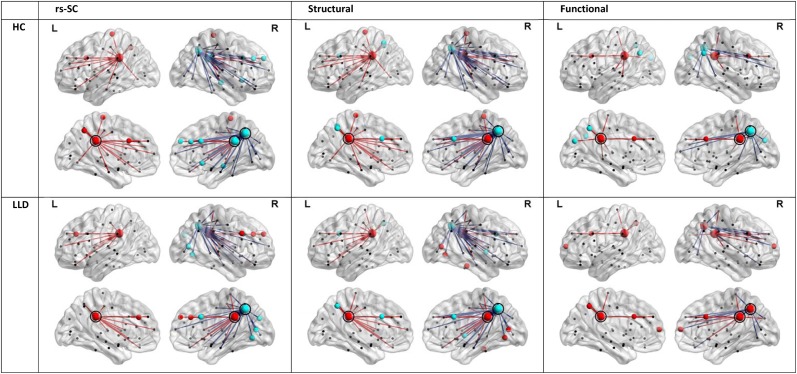
**Communities and connectivities for the resting-state structural connectome (rsSC), structural connectome, and functional connectome in healthy control (HC) and late-life depressed subjects (LLD), for nodes exhibiting significant group differences in the modular structure of rsSC shown in Figure 2**. The left posterior cingulate (circled in panels indicated “L”), right posterior cingulate (circled in panels indicated “R”) and the right precuneus (caudal and posterior to the right posterior cingulate in panels indicated “R”, also circled). For each diagnostic group, nodes that are coded the same color (either red or blue) form a community or module in the average tree for that group (computed by applying PLACE to the edge-wise average of all subjects' connectivity matrices in the same group, see methods section). Edges linked to the bilateral posterior cingulate are indicated in red, while edges linked to the right precuneus are in blue. For the functional connectome, edges were thresholded for the level of correlation >0.25. Of note, only the rsSC demonstrated significant differences in community structure. Visually, the pattern of associations in the rsSC are similar to those in Figure 2A for the left and right posterior cingulate (in that for HC there is a stronger association with ipsilateral precuneus), and for the right precuneus (in LLD there is a stronger association with occipital and posterior parietal cortices, consistent with a pattern of dorsal and anterior precuneus functional connectivity; also see discussion section).

In order to understand the implications of utilization differences between groups, we examined the global modular structure of the rsSC. To this end, we compared the community structure of the rsSC between groups by applying PLACE (with the mean rsSC of the HC group as the reference tree). We also applied PLACE to connectomes derived using data from a single imaging modality of either DTI alone or rs-fMRI alone. For functional connectome PLACE results, we followed the technique of (Schwarz and McGonigle, 2011) and analyzed the functional networks formed by positive (right-tail), negative (left-tail), and absolute correlation strengths across a range of thresholds (in increments of 0.05 until one or more of the functional networks become disconnected). Results revealed that there were no significant differences in modular structure when examining connectomes from a single modality. By contrast, after applying FDR correction (with a total of 82 comparisons), rsSC community structure was significantly altered for three regions with reduced consistency in LLD subjects: bilateral posterior cingulate, and the right precuneus (Figure 2). Visually, the bilateral posterior cingulate was more affiliated with posterior regions such as the precuneus in HC subjects, whereas in LLD bilateral posterior cingulate was more commonly associated with the anterior cingulate. With the right precuneus, results demonstrate a strong association with a limbic lobe module in HC subjects and a parietal lobe module in LLD subjects. Figure 3 visualizes the differential patterns of community affiliation and connectivity in the bilateral posterior cingulate and the right precuneus.

To determine whether standard community detection methods could yield similar results, we applied the modularity metric, Q to our sample. Again, there was no significant difference between groups using only structural or functional connectomes. However, there was a difference in community membership of the right fusiform gyrus using the rsSC with a significantly reduced *V* in LLD subjects compared to HC subjects (HC:0.801 ± 0.129, LLD:0.135 ± 0.209, *p* < 0.0001).

## Discussion

In this study, we adapted the recently-developed FSH mapping to construct the rsSC by projecting rsfMRI time series correlation information onto the whole brain tractography-derived structural connectome. To this end, we assumed that the rsfMRI correlation exhibits an exponential decay, subject to a rate constant, with respect to the “modulated” graph distance of the structural connectivity matrix. This allowed us to compute, as in the original FSH framework, a utilization matrix in order to determine whether the inclusion of a specific structural connection better explains the relationship between rsfMRI and the structural connectivity.

As expected, including the utilization matrix significantly increased the goodness of fit of the exponential decay model in both HC and LLD subjects. Network community structure of the rsSC using PLACE was altered in LLD subjects, particularly for regions associated with the posterior DMN comprising part of the limbic lobe and sub-regions of the parietal lobe. It is important to note that in contrast to our results with the rsSC, applying PLACE to the structural connectome or the functional connectome alone failed to yield any significant group differences (the same conclusion holds even when we used more conventional community detection methods, e.g., maximizing the Q modularity). This is suggestive of enhanced sensitivity to network modular structure differences in the integrated rsSC compared to connectomes derived from a single imaging modality.

The rsSC demonstrated altered community structure in a sub-network of nodes that belong to the posterior DMN and the limbic lobe. These nodes are notable for being associated with altered structural and functional connectivity in depression. The posterior cingulate is a part of the DMN which has been shown to be altered in depression (Greicius et al., 2007; Sheline et al., 2009), while as part of the posterior medial parietal cortex, the precuneus in recent years has been shown to play a central role in wide-ranging tasks including visuospatial imagery, episodic memory retrieval, and self-referential operations. Current evidence from functional studies supports a functional partition of the precuneus into an anterior division responsible for self-referential imagery, and a posterior division related to episodic memory retrieval (Cavanna and Trimble, 2006). Recent structural brain network studies using whole-brain tractography have also consistently established the precuneus as one of the many “hub” regions in the brain (i.e., regions with the most wide-spread connections to the rest of the brain; hub regions usually exhibit high degree centrality, i.e., serving as relay centers for information transfer across the brain) (van den Heuvel and Sporns, 2011; GadElkarim et al., 2012). Tracer studies in recent years have also established cortical connections between the precuneus and the frontal, the medial parietal, and the lateral parietal cortices (Cavanna and Trimble, 2006).

The findings of higher-degree associations between the precuneus and the lateral parietal cortex, and to some extent the sensorimotor regions have two main parallels with the known literature. First, the medial parietal cortex (including the precuneus) and lateral parietal cortex (especially the inferior parietal lobule) along with the medial prefrontal cortex have been shown to be primary regions activated during first person perspective tasks (Cavanna and Trimble, 2006). Secondly, a recent resting-state functional connectivity study of the precuneus has demonstrated a transitioning pattern of functional connectivity from the posterior and most ventral part of precuneus (greater connectivity with the medial superior frontal gyrus, orbitofrontal gyrus, anterior cingulate cortex, and parahippocampus) to the more dorsal and anterior part (greater connectivity with occipital and posterior parietal cortices and somatomotor cortex, among other regions) (Zhang and Li, 2012). Thus our observed rsSC community structure group differences suggest a pattern of posterior/ventral precuneus connectivity in the control group vs. a pattern of anterior/dorsal precuneus connectivity in the depressed group.

To conclude, using our novel multimodal integration technique FSH combined with PLACE, we detected a differential pattern of functional-structural connectome integration in late-life depressed subjects relative to controls.

### Conflict of interest statement

The authors declare that the research was conducted in the absence of any commercial or financial relationships that could be construed as a potential conflict of interest.
